# Non-Motor Symptoms as Markers of Disease Severity in Parkinson’s Disease: Associations Between Constipation, Depression, REM Sleep Behavior Disorder, and Motor Impairment

**DOI:** 10.3390/biomedicines13112704

**Published:** 2025-11-03

**Authors:** João Paulo Mota Telles, Júlia Haddad Labello, Lucas Camargo, Carla Pastora-Sesin, Anna Carolyna Gianlorenço, Felipe Fregni

**Affiliations:** 1 Department of Neurology, University of São Paulo, São Paulo 05403-010, Brazil; joao.telles@fm.usp.br (J.P.M.T.); julia.haddad@hc.fm.usp.br (J.H.L.); 2 Spaulding Neuromodulation Center and Center for Clinical Research Learning, Spaulding Rehabilitation Hospital, Harvard Medical School, Boston, MA 02115, USA; lcamargo@mgh.harvard.edu (L.C.); cpastorasesin@mgb.org (C.P.-S.); alepesteurgianlorenco@mgh.harvard.edu (A.C.G.); 3 Research Unit, The Regenerative Medicine Institute, Avenida Escazu, Torre Lexus, 4th Floor, San José 10203, Costa Rica; 4 Laboratory of Neuroscience and Neurological Rehabilitation, Physical Therapy Department, Federal University of São Carlos, São Carlos 13565-905, Brazil

**Keywords:** Parkinson disease, REM sleep behavior disorder, constipation, anosmia, depression

## Abstract

**Background:** This study aims to investigate the association between the presence and severity of non-motor symptoms (constipation, REM sleep behavior disorder [RBD], hyposmia, and depression) and the severity of motor impairment in Parkinson’s disease (PD). **Methods:** We used data from Parkinson’s Progression Markers Initiative (PPMI), comprising patients with established PD, prodromal PD, and healthy controls. Motor disability was evaluated with the MDS-UPDRS part III. Non-motor symptoms were assessed with standardized scales for constipation (MDS-UPDRS part I sub-item), depression (15-item GDS), RBD questionnaire (RBDQ), and hyposmia (UPSIT). The relationships between non-motor symptoms and motor severity were explored using linear regression models (adjusted for age/sex). **Results:** Constipation was significantly more prevalent in PD and prodromal PD and independently associated with greater motor severity in both groups (*p* < 0.001). Constipation also correlated with increased freezing and falls. Depressive symptoms were similar across groups, but in prodromal PD, higher GDS scores were associated with worse UPDRS III scores (*p* = 0.02), as well as higher freezing and fall scores. Hyposmia was strongly reduced in PD and prodromal PD compared with controls but was not independently associated with motor severity. Higher RBDQ scores were associated with worse motor impairment in PD, but not in prodromal PD after adjustment. **Conclusions:** Constipation and REM sleep behavioral disorder were independent correlates of worse motor severity in prodromal and established PD, whereas depressive symptoms predicted more severe parkinsonism only within the prodromal phase.

## 1. Introduction

Parkinson’s disease (PD), the second most common neurodegenerative disorder worldwide, is traditionally characterized by motor symptoms such as bradykinesia, rigidity, and resting tremor [1,2]. However, a broad spectrum of non-motor symptoms are also prominent features of the disease, including REM sleep behavior disorder (RBD), olfactory dysfunction, constipation, and mood disturbances such as depression and anxiety [3,4,5].

These non-motor symptoms’ pathophysiology probably involves a complex interplay between alpha-synuclein and other neurotransmitters, such as GABA, noradrenaline, serotonin, and acetylcholine [3,6]. Symptoms such as hyposmia, sleep disturbances and constipation may manifest years before motor impairment [7] or occur alongside the classic motor manifestations, significantly influencing patients’ clinical course and quality of life [8,9]. In this sense, these non-motor symptoms may serve both as direct consequences of PD pathology and as markers of diminished compensatory capacity, significantly shaping patients’ clinical course and quality of life.

Emerging evidence highlights the central role of glial dysfunction in Parkinson’s disease (PD). Microglia and astrocytes are essential for maintaining central nervous system homeostasis, but in the presence of α-synuclein aggregates they shift toward pro-inflammatory states, releasing cytokines, impairing synaptic function, and disrupting blood–brain barrier integrity [10].While glial cells also possess neuroprotective and repair capacities, such as phagocytosis of α-synuclein and trophic factor release, these compensatory responses appear to be gradually overwhelmed by chronic aggregation and inflammatory signaling.

Although non-motor symptoms such as constipation, depression, and sleep disorders are widely known phenomena in Parkinson’s disease, the role of them as predictive markers for the progression of the disease and compensatory failure is not clear enough. Determining whether these symptoms are a consequence of common mechanistic abnormalities, or more general impairment in the process of adaptation and resilience is important to elucidate the relationship with motor disease severity. In the present study, we explore the relationships between the occurrence and severity of major NMSs and motor impairment in Parkinson’s disease with an attempt to clarify their role as markers of a reduced compensatory capacity and progression of illness.

## 2. Methods

### 2.1. Study Cohort and Baseline Characteristics

This work uses data from the Parkinson’s Progression Markers Initiative (PPMI) [11], including patients with PD, patients with prodromal PD, and healthy controls. Collected data include demographics (age, sex, and ethnicity), clinical data on Parkinson’s disease symptoms and severity (motor assessments, non-motor symptoms, freezing of gait, and falls), and data on prodromal Parkinson’s disease symptoms (REM sleep behavioral disorder [RBD], constipation, depression, and hyposmia). If there were multiple assessments for the same variable, the earliest was used for analyses.

### 2.2. Symptom Assessment and Scales

Motor assessments were conducted using the Movement Disorders Society (MDS) Unified Parkinson’s Disease Rating Scale (UPDRS) part III [12]. Freezing of gait was evaluated using an ordinal scale: (i) Rare freezing when walking; may have start hesitation; (ii) Occasional freezing when walking; (iii) Frequent freezing; occasional falls from freezing; (iv) Frequent falls from freezing. Falls not related to freezing of gait were also graded in an ordinal scale: (i) None; (ii) Rare falling; (iii) Occasionally falls, less than once per day; (iv) Falls an average of once daily; (v) Falls more than once daily.

Evaluation of constipation followed the correspondent MDS-UPDRS part I scale sub-item: (0) Normal; (1) Slight; (2) Mild; (3) Moderate; (4) Severe. Depression was assessed using the 15-item geriatric depression scale (GDS) [13], and scores ≥ 5 were considered a positive depression screening, whereas scores ≥ 9 were considered moderate-to-severe depression. RBD was evaluated using the RBD Questionnaire [14] (0 to 13, with 0 meaning no symptoms). Polysomnographic and clinical confirmation of RBD was only available for prodromal PD and was therefore reported but not used in the analyses. Hyposmia was assessed using the University of Pennsylvania Smell Identification Test (UPSIT) percentiles, which are stratified according to age and sex.

### 2.3. Statistical Analysis

Continuous variables are described as mean (± standard deviation) or median (interquartile range), according to normality checks. Categorical variables are described as frequency (valid %). Comparisons of baseline characteristics between groups were performed with *t*-tests or chi-squared tests, as appropriate. Linear regressions were modelled to evaluate the association between prodromal symptoms and MDS-UPDRS part III scores, separating for patients with PD and prodromal PD. Multivariable analyses were adjusted for age and sex. Code is available at DOI: 10.5281/zenodo.17187305. All analyses were conducted using R (R Foundation for Statistical Computing 2022.07.1, Vienna, Austria, 2022).

## 3. Results

### 3.1. Baseline Characteristics

Baseline characteristics are shown in Table 1. Compared to controls, patients with PD were slightly older (63.5 ± 10.0 vs. 62.4 ± 11.3 years; *p* < 0.001) and more frequently male (61.0% vs. 58.5%; *p* < 0.001). Prodromal PD subjects were significantly older (67.7 ± 6.6 years) and had a lower proportion of males (43.9%; *p* < 0.001). Ethnic distribution varied among groups, but white individuals constituted the vast majority of PD (93.9%), prodromal PD (94.6%), and control cohorts (90.0%) (*p* < 0.001).

The mean UPDRS Part III score was substantially higher in PD patients (21.9 ± 10.2) compared to controls (1.5 ± 2.3) and prodromal PD individuals (4.5 ± 5.7), *p* < 0.001. Freezing of gait occurred exclusively in the PD group, affecting 17.5% of patients to varying degrees, while it was rare (1.5%) in prodromal PD and absent in controls (*p* < 0.001). Falls not related to freezing were also significantly more common in PD and prodromal PD groups, with around 20% of subjects in each reporting some fall episodes in the previous 12 months, compared to only 7.3% of controls (*p* < 0.001).

### 3.2. Constipation

Constipation, assessed using the UPDRS Part I scale, was more frequently reported in the PD and prodromal PD groups compared to controls (Table 2, *p* < 0.001). Among controls, 88.7% reported normal bowel function, versus 61.0% in PD and 64.8% in prodromal PD. Slight constipation was reported in 27.4% of PD patients and 25.0% of prodromal individuals, compared to 9.9% of controls. Moderate or greater constipation was uncommon in all groups but more frequent in PD and prodromal PD than in controls.

Table 3 presents the results of linear regression models evaluating the association between constipation severity and motor symptom severity, as measured by the total UPDRS Part III score, separately in PD and prodromal PD groups. In univariate analyses, higher constipation severity was positively associated with higher UPDRS III scores in both PD and prodromal PD cohorts. In the PD group, coefficients ranged from 2.17 (for slight constipation) to 21.79 (for severe constipation), with statistically significant associations for categories 1, 2, and 4. The continuous constipation variable also showed a significant association (coefficient: 1.55; *p* < 0.001). Similarly, in prodromal PD, significant associations were found for slight to moderate constipation (categories 1–3), and the continuous variable was also significantly associated with UPDRS scores (coefficient: 0.80; *p* < 0.001).

**Figure 1:**
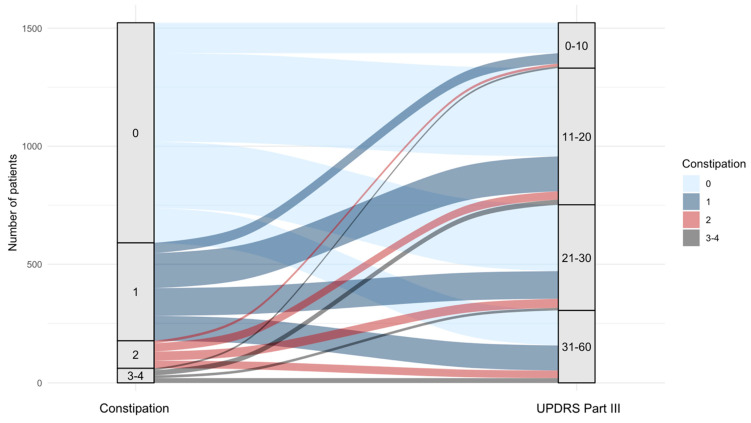
Distribution of total UPDRS Part III scores per constipation level. Constipation, rated according to the UPDRS Part I assessment from 0 (none) to 4 (Severe: I usually need physical help from someone else to empty my bowels), was significantly correlated with increasing UPDRS Part III scores (*p* < 0.001 for both prodromal and established PD). The figure shows the association between constipation and UPDRS Part III scores for all subjects: established PD, prodromal PD, and controls.

In multivariable models adjusted for age and sex, the positive associations between constipation severity and motor symptoms remained. In PD, slight and mild constipation (categories 1 and 2) and severe constipation (category 4) maintained statistical significance, with the latter showing a markedly higher coefficient (21.95; *p* < 0.001). Moderate constipation (category 3) approached but did not reach statistical significance. In prodromal PD, slight to moderate constipation (categories 1–3) remained significantly associated with higher UPDRS scores, while severe constipation (category 4) did not reach statistical significance. The continuous constipation score remained significantly associated with UPDRS III scores in both groups after adjustment. Figure 1 demonstrates the association between constipation scores and UPDRS Part III scores across all subjects.

Constipation was significantly correlated with higher scores for falls for patients with PD (Pearson’s product-moment coefficient + 0.063, *p* = 0.03) and prodromal PD (coefficient + 0.102, *p* < 0.001). Constipation was significantly correlated with higher scores for freezing of gait in the last 12 months for patients with PD (+0.07, *p* = 0.01), but not for prodromal PD (coefficient + 0.03, *p* < 0.11).

### 3.3. Depression

Regarding depressive symptoms, the mean Geriatric Depression Scale (GDS) scores were similar across groups (controls: 5.2 ± 1.2; PD: 5.5 ± 1.5; prodromal PD: 5.4 ± 1.3), and the frequency of GDS scores ≥5 was also comparable (82.2% in controls, 81.5% in PD, and 84.4% in prodromal PD; *p* = 0.056). However, a higher percentage of PD (4.2%) and prodromal PD (2.7%) participants had scores ≥9 compared to controls (1.5%; *p* = 0.004) (Table 4).

In the prodromal PD group, higher GDS scores were significantly associated with higher UPDRS III scores. This association remained statistically significant both in the univariate model (coefficient: 0.23; *p* = 0.01) and in the model adjusted for age and sex (coefficient: 0.21; *p* = 0.02), suggesting that greater depressive symptomatology is associated with increased motor impairment in the prodromal phase. In contrast, no statistically significant association was observed between GDS scores and UPDRS III scores in the PD group.

In patients with PD, higher total GDS scores were correlated with higher freezing scale scores (+0.076, *p* = 0.007), but not with falls (0.046, *p* = 0.10). In patients with prodromal PD, higher total GDS scores were correlated with higher freezing (+0.056, *p* = 0.008) and higher fall scores (+0.047, *p* = 0.03).

### 3.4. Hyposmia

Olfactory function, measured by the UPSIT percentile, was lower in PD (17.2 ± 21.8) and prodromal PD (13.0 ± 16.2) compared to controls (49.3 ± 30.1), with *p* < 0.001. In the PD group, no significant associations were found between UPSIT percentile and UPDRS part III scores (Table 5).

In the prodromal PD group, the univariate model indicated a statistically significant inverse association between UPSIT percentile and UPDRS score (coefficient: –0.017; *p* = 0.02), suggesting that lower olfactory function was associated with greater motor impairment. However, this association was not retained in the multivariable model adjusted for age and sex (coefficient: –0.007; *p* = 0.30).

In patients with PD, UPSIT percentile was correlated with falls (coefficient = 0.078, *p* = 0.04), but not with freezing of gait (−0.04, *p* = 0.30). In patients with prodromal PD, UPSIT percentage was inversely correlated with falls (−0.048, *p* = 0.03), but not with freezing of gait (−0.01, *p* = 0.55).

### 3.5. REM Sleep Behavioral Disorder

**Figure 2:**
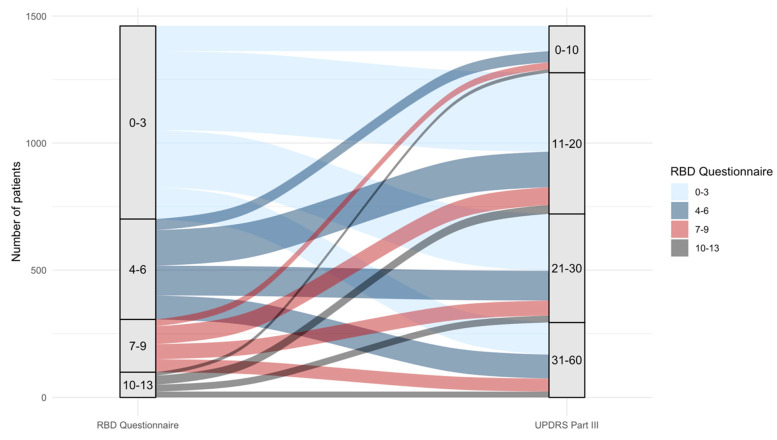
Distribution of total UPDRS Part III scores per RBD questionnaire scores. The REM sleep Behavioral Disorder (RBD) Questionnaire assessment from 0 to 13 (increasing scores mean increased symptoms), was significantly correlated with increasing UPDRS Part III scores in patients with established PD (*p* = 0.005). The figure shows the association between constipation and UPDRS Part III scores for all subjects: established PD, prodromal PD, and controls.

REM sleep behavior disorder (RBD) was assessed only in the prodromal group, where 67.3% had PSG-confirmed RBD and 56.7% had a clinical diagnosis. RBD questionnaire scores increased across groups (controls: 2.5 ± 2.4; PD: 4.1 ± 3.0; prodromal PD: 5.6 ± 4.1; *p* < 0.001) (Table 6). In the PD group, higher RBD scores were significantly associated with greater UPDRS Part III scores. This relationship remained significant in both univariate (coefficient: 0.27; *p* = 0.002) and multivariable models adjusted for age and sex (coefficient: 0.25; *p* = 0.005). Figure 2 demonstrates the association between RBD scores and UPDRS Part III scores across all subjects.

In the prodromal PD group, RBD questionnaire scores were also associated with UPDRS scores in the univariate model (coefficient: 0.13; *p* < 0.001). However, this association did not remain statistically significant after adjustment for age and sex in the multivariable model (coefficient: 0.05; *p* = 0.08).

Higher total RBD questionnaire scores were significantly correlated with freezing of gait in patients with PD (+0.084, *p* = 0.003), but not prodromal PD (+0.025, *p* = 0.23). The questionnaire scores were also correlated with falls, both in patients with PD (+0.07, *p* = 0.01) and prodromal PD (*p* = 0.03).

## 4. Discussion

In this study from a cohort of over 7000 subjects, we observed that constipation is correlated not only with higher UPDRS part 3 scores in both established and prodromal PD, but also with worse fall risk and freezing of gait scores. RBD symptom burden was also associated with worse motor severity in PD and with falls in both groups. Depressive symptoms seem to be related to greater motor severity only in prodromal PD and to gait issues in both groups. Hyposmia was not associated with motor scores.

Constipation is a prevalent non-motor symptom that affects not only PD patients but the general elderly population, mainly women [15]. This condition has a direct impact on patients’ quality of life, limits physical functioning, and is associated with poorer mental health [16,17]. The descending dopaminergic innervation to the spinal cord originates from A11 nuclei of the posterior hypothalamus, releasing dopamine (DA) into the lumbosacral defecation center in L6-S1 regions, targeting dopamine D2-like receptors, to increase the colorectal motility [18].

Sanger et al. (2016) [19] suggest that the dopaminergic pathway could explain the pathogenesis of constipation on PD, as the neurodegeneration of central areas responsible for dopaminergic input to the lumbosacral defecation area are affected in those patients. In our analysis, constipation emerged as one of the most robust non-motor correlations of motor severity.

Moreover, constipation was significantly associated with freezing of gait in PD and with greater fall risk in both PD and prodromal PD, which demonstrate its relevance not only to overall motor burden but also to gait-measuring outcomes. This is in line with previous findings by Yu et al. (2018) [20], who observed that the PD group with constipation were older, had longer disease durations and more severe motor and other non-motor symptoms.

Depression in PD is hypothesized to arise from neurodegenerative changes that extend beyond the substantia nigra pars compacta and involve mesolimbic and mesocortical pathways that originate in the ventral tegmental area [21]. In our analysis, depressive symptoms measured by the GDS were independently associated with greater motor severity only in prodromal PD, after adjusting for age and sex. The analysis of patients with established PD might have been limited by a lower sample size compared to prodromal. Across both PD and prodromal PD, higher GDS scores correlated with gait-related outcomes, including freezing of gait, and with falls in prodromal PD. These findings align with previous longitudinal analysis showing that depression in early PD predicts faster motor decline [22] and with evidence linking mood symptoms to impaired attentional performance that may contribute to gait instability and fall risk [23].

RBD is a recognized prodromal marker of α-synucleinopathies, reflecting early degeneration in brainstem regions responsible for REM atonia, particularly the sublaterodorsal tegmental nucleus, with frequent extension to locomotor control structures such as the pedunculopontine and cuneiform nuclei [24].

Although the association of RBD questionnaire scores with motor severity was significant in PD after adjustment, it did not persist in prodromal PD once demographics were considered, possibly reflecting cohort heterogeneity and the inclusion of individuals with idiopathic RBD who may not convert to PD within the study period. These patterns are consistent with previous findings showing that PD patients with RBD have faster motor progression, earlier onset of postural instability, and increased fall risk [25].

Although olfactory dysfunction was markedly present in both PD and prodromal PD compared with controls (*p* < 0.001), there were no consistent associations between hyposmia and motor severity, falls, and freezing of gait. This suggests that olfactory dysfunction is not correlated to motor symptoms in a dose-dependent fashion, such as we have seen in constipation. On the other hand, this may be partially derived from the limitations of smell tests [26], since olfaction is a chemical sense with a myriad of possibilities, making it extremely difficult to assess objectively.

### 4.1. Compensatory Salutogenesis in Parkinson’s Disease

From a salutogenic perspective, the non-motor symptoms observed here—constipation, depression, and REM sleep behavior disorder (RBD)—can be understood as markers of reduced compensatory reserve, which might reflect the nervous system’s inability to buffer the effects of α-synuclein pathology and neuroinflammation.

### 4.2. Constipation

Constipation emerged as one of the most robust non-motor correlates of motor severity in both established and prodromal PD. Beyond impaired dopaminergic modulation of the defecation center, recent evidence highlights gut dysfunction as a potential trigger for PD pathology itself. Early enteric nervous system changes may promote constipation and inflammation, fostering α-synuclein aggregation in the gut that subsequently spreads to the brain via vagal pathways [27]. Within a compensatory framework, constipation might therefore reflect both direct gut–brain pathology and the system’s failure to maintain adaptive regulation of peripheral–central circuits under inflammatory stress.

### 4.3. Depression

Depression was independently associated with greater motor severity in prodromal PD. Mechanistically, PD-associated depression is increasingly linked not only to dopaminergic but also to noradrenergic and serotonergic dysfunction. These systems are particularly vulnerable to inflammatory processes, with neuroinflammation disrupting neurotransmitter balance and impairing mood regulation [28]. Viewed through a salutogenic lens, depression in prodromal PD could signal the collapse of compensatory cross-talk between monoaminergic pathways and glial support systems. These alterations were significant in the prodromal phase and not in the established PD, which might be because these prodromic patients with depression have more prominent support system failure, which reaches a common final pathway in patients with established PD.

### 4.4. REM Sleep Behavior Disorder (RBD)

RBD was associated with worse motor severity and increased falls, reinforcing its role as a marker of aggressive PD progression. New evidence indicates that individuals with isolated RBD (iRBD) exhibit glymphatic dysfunction, impairing the clearance of neurotoxic proteins and strongly predicting phenoconversion to PD [29]. This suggests that RBD reflects not just localized brainstem degeneration but a broader failure of waste-clearance and homeostatic systems essential for neural resilience. In salutogenic terms, the breakdown of glymphatic compensation might reflect α-synuclein accumulation and contributes to loss of inhibitory motor control.

### 4.5. Cross-Condition Evidence for Compensatory Failure

Similar compensatory breakdowns are observed in other chronic conditions. In knee osteoarthritis (KOA), activity-related pain is linked to reduced cortical inhibition, elevated beta oscillations, and defective conditioned pain modulation (CPM), demonstrating a lack of compensatory inhibitory control. By contrast, pain at rest relates more to psychological factors without these neural deficits [30]. This dissociation shows how symptom severity increases when inhibitory resources fail under activity demands.

Likewise, a meta-analysis of heart rate variability (HRV) in pain trials revealed that interventions can restore vagal activity and parasympathetic dominance, reflecting improved autonomic compensation. Conversely, lower HRV signals autonomic failure and greater pain dysregulation, and body mass index (BMI) moderates the capacity for autonomic recovery [31]. HRV thus acts as a biomarker of compensatory reserve, conceptually parallel to constipation, depression, and RBD in PD.

### 4.6. Integrative Perspective

Together, constipation, depression, and RBD represent interconnected signs of diminished compensatory capacity across peripheral, limbic, and brainstem systems. Each highlights a specific pathway—gut–brain integration, monoaminergic mood regulation, and glymphatic clearance—where glial and inflammatory mechanisms can no longer sustain homeostasis. Hyposmia, by contrast, appears more as a static marker of α-synuclein pathology than a dynamic indicator of compensation failure. Importantly, evidence from osteoarthritis and HRV pain studies reinforces that across conditions, symptoms intensify when compensatory systems fail, whether inhibitory, autonomic, or glial. Recognizing non-motor features in PD as mechanistic reflections of salutogenesis breakdown therefore provides a unifying framework for their relationship with motor severity, prognosis, and therapeutic targeting.

### 4.7. Strengths and Limitations

This work presents several limitations. The scales used to quantify non-motor symptoms, although validated and widely used, may not capture subtleties of these complex manifestations. Secondly, there are numerous confounders that can affect symptoms such as constipation and hyposmia, even though the large population tends to soften this sort of bias. The cross-sectional nature of the study limits the ability to infer causality, and prospective studies could evaluate this association in more depth. Finally, data for non-motor symptoms were not uniformly collected for all patients, which limited the analyses to a subset of the entire population. Nevertheless, the study also presents several strengths, including the large sample size and the marked differences in prevalence of non-motor symptoms between PD patients and controls, demonstrating the validity of the measurements.

## 5. Conclusions

Constipation and REM sleep behavioral disorder severity were significantly associated with worse motor scores in patients with both established and prodromal PD. Depression severity was associated with worse motor scores in patients with prodromal, but not established PD. Hyposmia was not associated with motor scores in either cohort.

## Figures and Tables

**Table 1:** Patient characteristics.

	Controls (*n* = 440)	PD (*n* = 1919)	Prodromal PD (*n* = 4964)	*p*-Value
Sex (Male)	257 (58.5%)	1168 (61.0%)	2152 (43.9%)	<0.001
Age	62.4 (±11.3)	63.5 (±10.0)	67.7 (±6.6)	<0.001
Ethnicity				
White	395 (90.0%)	1795 (93.9%)	4636 (94.6%)	<0.001
Hispanic/Latino	14 (3.2%)	118 (6.2%)	234 (4.8%)	<0.001
Black	27 (6.2%)	34 (1.8%)	71 (1.4%)	<0.001
Asian	8 (1.8%)	32 (1.7%)	55 (1.1%)	0.12
Other	15 (3.5%)	67 (3.5%)	124 (2.5%)	0.02
Total UPDRS Part III Scores	1.5 (±2.3)	21.9 (±10.2)	4.5 (±5.7)	<0.001
Freezing of Gait (Past 12 months)				<0.001
Rare freezing when walking; may have start hesitation	0	115 (9.4%)	29 (1.3%)	
Occasional freezing when walking	0	68 (5.5%)	5 (0.2%)	
Frequent freezing; occasional falls from freezing	0	29 (2.4%)	0	
Frequent falls from freezing	0	3 (0.2%)	0	
Falls not related to freezing (Past 12 months)				<0.001
None	240 (92.7%)	952 (77.6%)	1810 (79.9%)	
Rare falling	19 (7.3%)	239 (19.5%)	427 (18.9%)	
Occasionally falls, less than once per day	0	30 (2.4%)	27 (1.2%)	
Falls an average of once daily	0	4 (0.3%)	0	
Falls more than once daily	0	2 (0.2%)	0	

Data are presented as mean (±standard deviation) or frequency (valid %). *p*-values refer to ANOVA or chi-squared tests, as appropriate. PD = Parkinson’s Disease.

**Table 2:** Prodromal symptoms.

	Controls	PD	Prodromal PD	*p*-Value
Constipation *				<0.001
(0) Normal	305 (88.7%)	940 (61.0%)	1622 (64.8%)	
(1) Slight	34 (9.9%)	422 (27.4%)	627 (25.0%)	
(2) Mild	3 (0.9%)	116 (7.5%)	174 (6.9%)	
(3) Moderate	2 (0.6%)	60 (3.9%)	78 (3.1%)	
(4) Severe	0	3 (0.2%)	4 (0.2%)	
Depression				
Total GDS scores	5.2 (±1.2)	5.5 (±1.5)	5.4 (±1.3)	0.13
GDS ≥ 5	281 (82.2%)	1209 (81.5%)	2108 (84.4%)	0.056
GDS ≥ 9	5 (1.5%)	63 (4.2%)	67 (2.7%)	0.004
Hyposmia				
UPSIT Percentile	49.3 (±30.1)	17.2 (±21.8)	13.0 (±16.2)	<0.001
REM sleep behavioral disorder				
PSG-confirmed RBD	NA	NA	565 (67.3%)	0
Clinical RBD diagnosis	NA	NA	808 (56.7%)	0
Total RBD Questionnaire Scores	2.5 (±2.4)	4.1 (±3.0)	5.6 (±4.1)	<0.001

Data are presented as mean (±standard deviation) or frequency (valid %). *p*-values refer to ANOVA or chi-squared tests, as appropriate. * Constipation is rated according to the UPDRS Part I assessment: 0: Normal: No constipation. 1: Slight: I have been constipated. I use extra effort to move my bowels. However, this problem does not disturb my activities or my being comfortable. 2: Mild: Constipation causes me to have some troubles doing things or being comfortable. 3: Moderate: Constipation causes me to have a lot of trouble doing things or being comfortable. However, it does not stop me from doing anything. 4: Severe: I usually need physical help from someone else to empty my bowels. GDS = Geriatric Depression Scale. NA = not available. PD = Parkinson’s Disease. PSG = polysomnography. RBD = REM sleep behavioral disorder. UPDRS = Unified Parkinson’s Disease Rating Scale. UPSIT = University of Pennsylvania Smell Identification Test.

**Table 3:** Association of constipation and UPDRS part III scores.

	Coefficient	Std. Error	*p*-Value
**Parkinson’s Disease**			
Univariate			
1	2.17	0.59	<0.001
2	3.3	0.99	<0.001
3	2.53	1.35	0.06
4	21.79	5.82	<0.001
Continuous	1.55	0.32	<0.001
Multivariable			
1	1.77	0.59	0.002
2	2.68	0.98	0.006
3	2.19	1.33	0.10
4	21.95	5.74	<0.001
Continuous	1.32	0.32	<0.001
**Prodromal PD**			
Univariate			
1	0.91	0.27	<0.001
2	1.53	0.45	<0.001
3	2.26	0.66	<0.001
4	4.19	2.82	0.14
Multivariable			
1	0.67	0.26	0.009
2	1.18	0.44	0.007
3	2.08	0.64	0.001
4	3.66	2.72	0.18
Continuous	0.66	0.14	<0.001

Linear regressions with total UPDRS part III scores as the dependent variable. Multivariable analyses are adjusted for age and sex. PD = Parkinson’s Disease. UPDRS = Unified Parkinson’s Disease Rating Scale.

**Table 4:** Association of depression and UPDRS part III scores.

	Coefficient	Std. Error	*p*-Value
**Parkinson’s Disease**			
Univariate			
Total GDS	0.18	0.17	0.31
Multivariable			
Total GDS	0.27	0.17	0.11
**Prodromal PD**			
Univariate			
Total GDS	0.23	0.09	0.01
Multivariable			
Total GDS	0.21	0.09	0.02

Linear regressions with total UPDRS part III scores as the dependent variable. Multivariable analyses are adjusted for age and sex. GDS = Geriatric Depression Scale. PD = Parkinson’s Disease. UPDRS = Unified Parkinson’s Disease Rating Scale.

**Table 5:** Association of hyposmia and UPDRS part III scores.

	Coefficient	Std. Error	*p*-Value
**Parkinson’s Disease**			
Univariate			
UPSIT percentage	−0.02	0.02	0.23
Multivariable			
UPSIT percentage	−0.03	0.02	0.11
**Prodromal PD**			
Univariate			
UPSIT percentage	−0.017	0.007	0.02
Multivariable			
UPSIT percentage	−0.007	0.007	0.30

Linear regressions with total UPDRS part III scores as the dependent variable. Multivariable analyses are adjusted for age and sex. PD = Parkinson’s Disease. UPDRS = Unified Parkinson’s Disease Rating Scale. UPSIT = University of Pennsylvania Smell Identification Test.

**Table 6:** Association of REM sleep behavioral disorder and UPDRS part III scores.

	Coefficient	Std. Error	*p*-Value
**Parkinson’s Disease**			
Univariate			
RBD Questionnaire	0.27	0.09	0.002
Multivariable			
RBD Questionnaire	0.25	0.09	0.005
**Prodromal PD**			
Univariate			
RBD Questionnaire	0.13	0.03	< 0.001
Multivariable			
RBD Questionnaire	0.05	0.03	0.08

Linear regressions with total UPDRS part III scores as the dependent variable. Multivariable analyses are adjusted for age and sex. PD = Parkinson’s Disease. UPDRS = Unified Parkinson’s Disease Rating Scale. UPSIT = University of Pennsylvania Smell Identification Test.

## Data Availability

The original data presented in the study are openly available in Parkinson’s Progression Markers Initiative at http://ppmi-info.org/.
